# Agenesis of the Corpus Callosum in a Patient With Schizophrenia

**DOI:** 10.7759/cureus.16058

**Published:** 2021-06-30

**Authors:** Jacob B Rosewater, Michelle Zaydlin, Stephen A McLeod-Bryant

**Affiliations:** 1 Psychiatry and Behavioral Sciences, University of Miami Miller School of Medicine, Jackson Memorial Hospital, Miami, USA

**Keywords:** psychosis, agenesis of corpus callosum, psychiatry & mental health, psychiatry, schizophrenia, neuropsychiatry, case report, agenesis, corpus callosum

## Abstract

The aim of this study is to report the case of a 25-year-old male with a history of schizophrenia who presented involuntarily to the psychiatric emergency department (ED) due to worsening agitation, paranoia, and disorganized behavior concerning a psychotic episode. During medical clearance to rule out acute organic causes of altered mental status prior to admission, the patient was found to have agenesis of the corpus callosum (ACC) on CT of the brain. ACC is a rare congenital disorder characterized by the partial or complete absence of the commissural pathway that connects the two cerebral hemispheres. This case presents a thought-provoking incidental anatomical finding in a patient with an acute exacerbation of chronic schizophrenia and allows for further discussion about the prevalence of undiagnosed malformations and possible underlying genetic contributions in patients with chronic mental illness.

## Introduction

The corpus callosum, a large white matter tract, is the main commissural pathway that connects the two cerebral hemispheres. This brain structure has an important role in the integration of information between the two hemispheres of the brain and plays a primary role in cognitive functioning. Agenesis of the corpus callosum (ACC) is a rare congenital anomaly that involves the partial or complete absence of the corpus callosum; ACC can be isolated, occurring alone, or complex, coexisting with other congenital abnormalities. Additionally, agenesis can be complete, indicating the absence of all components of the corpus callosum, or partial, indicating the presence of a short remnant. Dysgenesis and hypogenesis are alternate terms sometimes used to describe partial ACC [1]. 

While the true prevalence of ACC may be difficult to measure [1], one study which examined a population-based registry of birth defects from 1983 to 2003 estimated the prevalence of ACC to be 1.8 per 10,000 births [2]. Other studies have reported the incidence of ACC as falling between 0.05% and 0.7% of the general population with a male predominance [3]. Malformations of the corpus callosum are observed in a variety of conditions that disrupt early cerebral development, including chromosomal and metabolic disorders as well as intrauterine infections and exposure to teratogens [4]. Additional risk factors for ACC include prematurity, chromosomal abnormalities and accompanying musculoskeletal, cardiac and central nervous system (CNS) malformations, as well as infants with a chromosomal disorder born to mothers with advanced maternal age ≥40 years [2].

In addition to a diverse diagnostic presentation, ACC also has a wide range of clinical presentations. In some patients, ACC may be entirely asymptomatic or with only mild intellectual difficulties, including deficits in visual processing, higher-order language functions, and social delays apparent only on detailed psychometric testing. While there is no well-defined syndromic classification of corpus callosum pathology, the current literature suggests an association between neuropsychiatric symptoms and degeneration or ACC [3]. These include comorbidities such as attention deficit or autism spectrum disorders as well as disruption of emotional processing and predisposition to psychosis, with many patients experiencing delusions specifically [2-3].

## Case presentation

This is the case of a 25-year-old male with a past psychiatric history of schizophrenia who presented to the behavioral health emergency department (ED) under an involuntary hold for psychiatric evaluation due to worsening psychosis, with symptoms including persecutory delusions, auditory hallucinations and aggressive behaviors, leading to concern for risk of harm to self and others. On arrival, the patient was observed appearing agitated and paranoid with disorganized and threatening behavior, including aggression toward law enforcement officers who escorted the patient to the ED. The patient appeared to be confused and had difficulty providing clear information to the treatment team upon initial evaluation in the psychiatric ED. The patient’s medical and surgical history was remarkable for right retinal detachment status-post seven eye surgeries, left eye blindness due to accidental traumatic injury during childhood, and chronic disc herniation with associated chronic back pain.

As the patient was unable to provide a clear history to the treatment team, his father was contacted for collateral information. On interview, he indicated that due to extenuating circumstances, the patient had been unable to obtain his medications for chronic back pain, including oxycodone 20 mg oral (PO) four times daily and transdermal fentanyl patch 50 μg, one patch applied to the skin daily. The father stated that the last dose was taken one week prior to the presentation. It was suspected that inadequate pain control contributed to nonadherence with psychotropic medication regimens including antipsychotic medication for psychosis as well as benzodiazepines for anxiety with alprazolam 1 mg PO three times per day. The state’s online prescription drug monitoring program corroborated this information. In combination, this all resulted in an acute exacerbation of chronic psychosis with aggressive and disorganized behavior, leading to the patient’s psychiatric evaluation.

Due to the patient’s acute presentation in the context of abrupt discontinuation of opioids and benzodiazepines, the patient was sent to the medical ED for a more extensive medical evaluation. In the ED, the patient remained verbally aggressive and combative, prompting the administration of lorazepam 1 mg PO.

**Figure 1:**
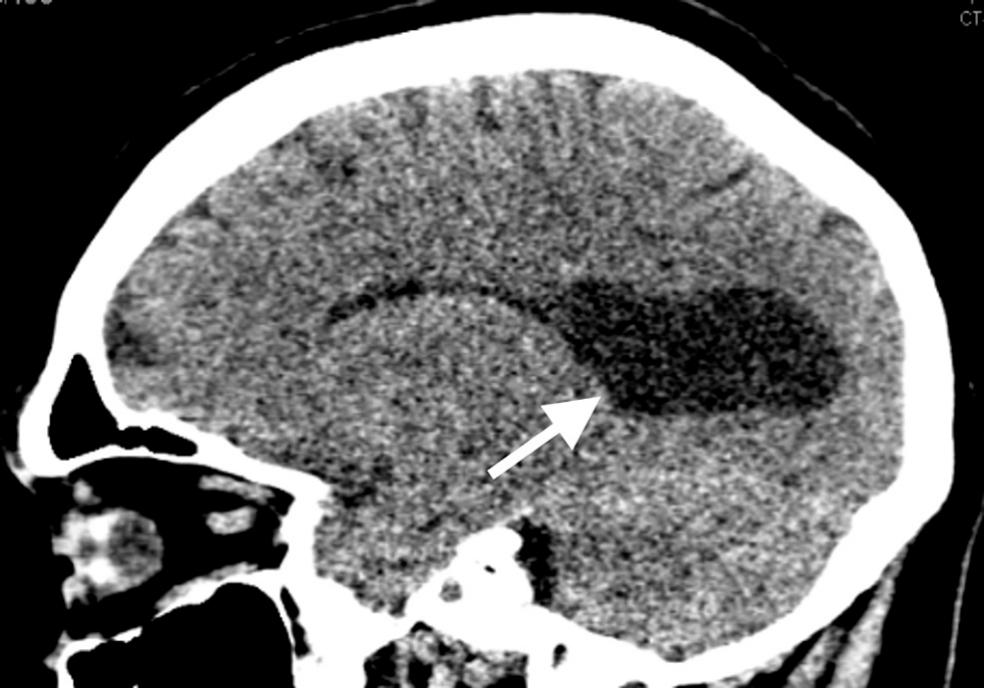
CT brain without contrast revealing colpocephaly (arrow) and absence of the corpus callosum.

On examination, the patient was hemodynamically stable, and the physical exam was within normal limits other than the aforementioned visual deficits; the patient had been previously reported as retaining only approximately 25% of the vision in his right eye. Laboratory examination was grossly unremarkable. Additionally, CT of the brain without contrast was completed for evaluation of altered mental status, confusion, and agitation (Figure 1). The radiologist’s read deemed the imaging significant for “evidence of colpocephaly with pronounced enlargement of the trigone of the lateral ventricles in comparison with the frontal horns, as well as the parallel orientation of the lateral ventricles. In addition, a normal corpus callosum is not clearly identified; this constellation of findings suggests agenesis/dysgenesis of the corpus callosum.” MRI of the brain was offered for further characterization but was not completed during an inpatient admission, as there were no acute concerns regarding this finding on the patient’s presentation.

Following medical clearance, the patient was transferred back to the behavioral health ED. Upon presentation, the patient was calm and cooperative but still grossly psychotic and disorganized, stating "I am a commander of the army where we fight monsters and submarines." The patient then signed voluntarily for admission to acute inpatient psychiatry.

On admission, the patient was noted to be isolative but alert and oriented to person, place, and time. In an initial interview with the primary inpatient psychiatric team, the patient demonstrated poor insight into the current situation and events leading to hospitalization. He demonstrated paranoid delusions, reporting that there was a "rotor" that was used to monitor his whereabouts. He also demonstrated grandiosity discussing how he had been part of the Central Intelligence Agency (CIA) since the age of three and had traveled the world extensively as part of the military until age four or five. The patient used bizarre neologisms and was tangential at times. At this time, no psychotropic medications were started due to patient refusal and his voluntary, capacitated admission status. However, as the patient had abruptly discontinued home medications of alprazolam and oxycodone, he was placed on the Clinical Institute Withdrawal Assessment for Alcohol (CIWA) and Clinical Opiate Withdrawal Score (COWS) protocols to monitor for symptoms of benzodiazepine and opioid withdrawal. The only present physical complaint was his chronic back pain.

On interview the following day, the patient continued exhibiting poor insight into his illness, the purpose of treatment, and the risks of treatment refusal. He was then converted to an involuntary and incapacitated status with his father signing as a healthcare proxy (HCP). With permission from the HCP, the patient was restarted on a home medication regimen of sodium valproate 500 mg PO at bedtime for mood stabilization and risperidone 1 mg PO at bedtime for psychosis. After a later discussion with the patient's father regarding prior response to risperidone and paliperidone palmitate, the decision was made to discontinue sodium valproate and optimize risperidone dosage with a plan to convert the patient to paliperidone palmitate intramuscular (IM) injections to be administered monthly for improved medication adherence. Risperidone was titrated to 2 mg PO daily with good effect and was tolerated well, thus the patient received an IM injection of 234 mg of paliperidone palmitate on day four of hospitalization with the plan to receive a second loading dose injection of 156 mg four to 10 days after initial injection with a continued monthly dose for maintenance.

The patient was also seen by the acute pain consult team, who recommended the following regimen for the patient's chronic pain: celecoxib 200 mg PO daily, diclofenac gel four times daily, gabapentin 300 mg PO three times daily, which was further increased to 600 mg three times per day, baclofen 20 mg PO twice daily as needed, and oxycodone 30 mg PO every six hours as needed. The patient requested one oxycodone dose daily with adequate control of pain. Utilizing the CIWA and COWS protocols, no acute withdrawal symptoms were noted throughout admission; however, it is notable that the patient had run out of prescribed benzodiazepines and opioids about one week prior to admission. He additionally complained of anxiety during admission, for which he received lorazepam 0.5 mg once daily as needed for several days, which was discontinued prior to discharge, once other psychotropic medications were optimized based on symptom improvement.

Throughout the rest of his hospitalization, the patient was cooperative with the treatment plan and did not pose any management problems. The patient did not require any emergency treatment orders for behavioral issues. After eight days of inpatient treatment, the patient exhibited and endorsed improvements in behavioral and psychotic symptoms, denied any suicidal or homicidal thoughts, had returned to his baseline level of functioning, and was therefore deemed psychiatrically stable for discharge. Prior to discharge, a safety plan including continued medication adherence, outpatient psychiatry follow-up, and abstinence from substances was discussed at length with the patient and he expressed agreement and understanding of this plan.

## Discussion

While ACC has been previously described in patients with neuropsychiatric symptoms, the number of reports regarding corpus callosal malformation and psychosis remains limited and the underlying pathophysiologic mechanism is not well understood [3]. There is still no well-defined syndromic classification of corpus callosum pathologies, however, it has been associated with many chromosomal and metabolic disorders, CNS and somatic abnormalities, and intrauterine exposures to teratogens and infections [4-8].

In practice, it can be difficult to identify with certainty the presence or absence of the corpus callosum. In this case report, it appears that the patient underwent CT of the brain in the ED in order to rule out possible secondary causes of altered mental status and psychotic behavior. While MRI to further characterize the patient’s corpus callosal degeneration was offered and not completed, the final report from the radiologist describes CT findings highly suggestive of ACC, namely colpocephaly, or dilatation of the atria and occipital horns of the lateral ventricles [1]. This patient had no known previous imaging for comparison and otherwise may have gone undiagnosed with ACC for the rest of his life.

This patient has a past medical history of right retinal detachment as well as additional CT findings of irregular globe morphologies suggestive of bilateral colobomas. Colobomas are congenital abnormalities consisting of gaps or holes in ocular tissues resulting from the failure of the embryonic fissure to close during the fifth to seventh week of fetal life. Colobomas are often associated with genetic defects and are found in syndromic forms, such as in CHARGE (Coloboma, Heart defect, Atresia choanae, Retarded growth and development, Genital hypoplasia, and Ear anomalies/deafness) and COACH (Cerebellar vermis hypoplasia, Oligophrenia, Ataxia, Coloboma, and Hepatic fibrosis) syndromes [9-10]. Perhaps it is worth considering whether this patient may have an undiagnosed genetic or chromosomal disorder. As previously discussed, ACC has been associated with chromosomal, genetic, and other disorders, and schizophrenia spectrum disorders are proposed to have a substantial underlying genetic risk as well [11-13].

## Conclusions

Agenesis of the corpus callosum accompanied by a psychotic disorder such as schizophrenia remains a relatively under-studied phenomenon. While there are case reports and literature reviews published on chronicle neuropsychiatric-associated corpus callosal malformations, there is a lot that we do not understand about the underlying pathophysiologic mechanisms leading to significant symptomatology. This case presents an opportunity for interesting discussions regarding possible underlying genetic or chromosomal disorders in patients with psychotic disorders as well as whether ACC in patients with psychosis may be more prevalent than previously thought.

Considering the unknown accuracy of estimated incidence and prevalence of ACC in combination with an unknown number of individuals with ACC thought to be largely asymptomatic, future studies on the implications of such cases may be worthwhile. Perhaps one-day novel therapeutic modalities may be tailored to various underlying anatomical brain abnormalities in patients with psychosis.
